# Supplementary material for: Met-Flow, a strategy for single-cell metabolic analysis highlights dynamic changes in immune subpopulations
Source: Commun Biol. 2020 Jun 12;3:305. doi: 10.1038/s42003-020-1027-9 (PMC7292829; doi:10.1038/s42003-020-1027-9)
Supplement: Supplementary file 1 — Supplementary Information [file 42003_2020_1027_MOESM1_ESM.pdf]

Met-Flow: Supplementary Figures

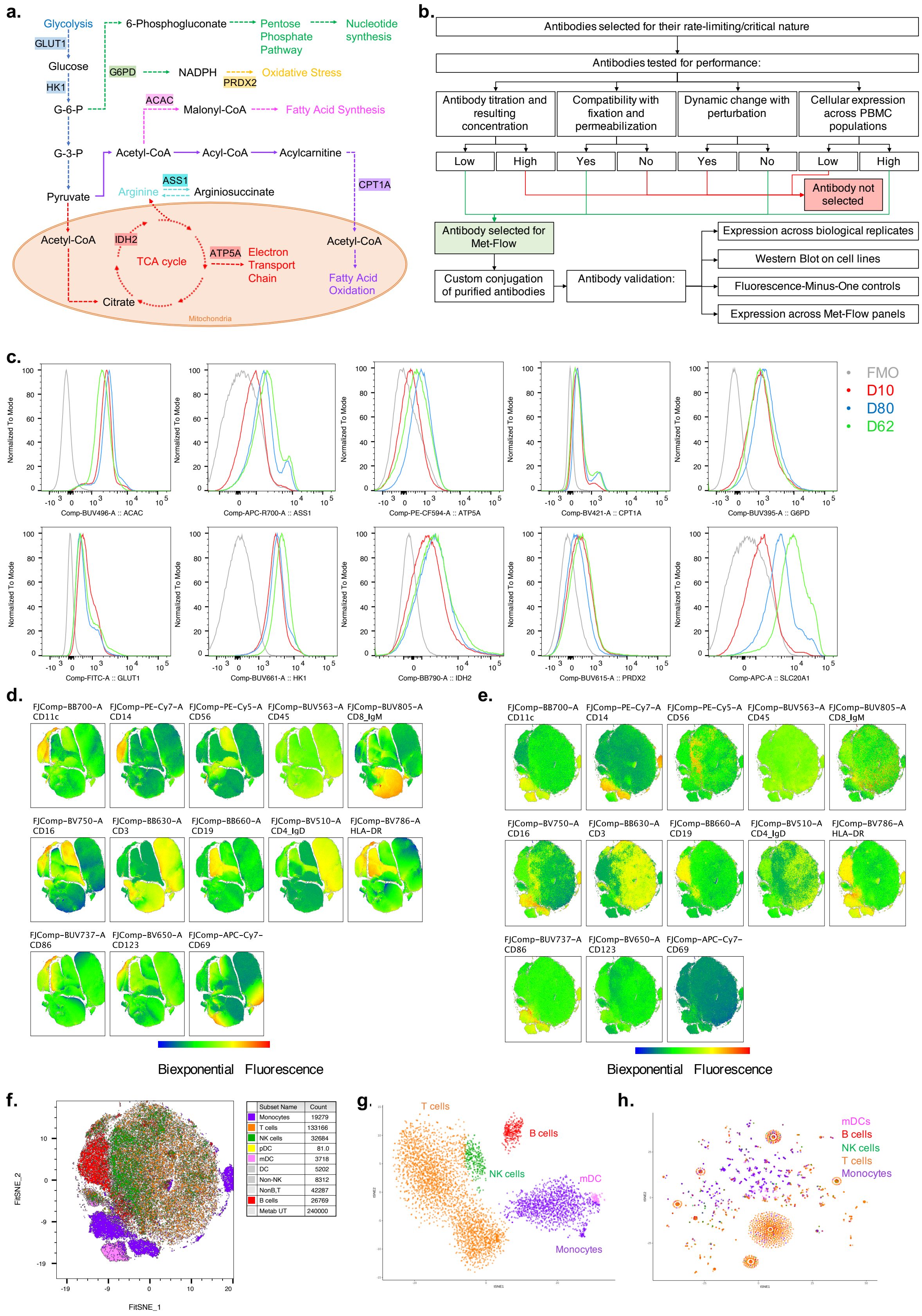

Supplementary Figure 1. Metabolic pathways and proteins analyzed using Flow Cytometry. (a) Ten specific metabolic enzymes were selected, which are critical and rate-limiting in each

distinct pathway. These represent glucose metabolism by the glucose transporter (GLUT1), Hexokinase 1 (HK1), the pentose phosphate pathway by Glucose-6-Phosphate Dehydrogenase (G6PD), oxidative stress regulation by Peroxiredoxin 2 (PRDX2), phosphate import by Solute carrier family 20 member 1 (SLC20A1), fatty acid metabolism by Acetyl-Co-A Carboxylase Alpha (ACAC) and Carnitine-Palmitoyl Transferase 1A (CPT1A), urea cycle by Arginosuccinate-Synthetase 1 (ASS1), and oxidative phosphorylation by mitochondrial isocitrate dehydrogenase (IDH2) and ATP synthase F1 subunit alpha (ATP5A). (b) The antibodies were optimized for Met-Flow by testing their performance, including antibody titration, compatibility with fixation and permeabilization buffers, degree of divergent expression across PBMC subsets, as well as the ability to measure changes in the protein levels with perturbations. (c) Expression of each metabolic protein in n=3 healthy donors stained with all antibodies, compared to the fluorescence-minus-one (FMO) control, stained with all except the corresponding antibody. (d) Heatmap of phenotypic markers to identify immune populations on the FitSNE projection of immune markers with metabolic protein level representing n=12 donors, (e) and on the projection of metabolic proteins only. (f) Overlay of conventionally gated immune populations. (g) tSNE projection of scRNAseq in purified PBMC using around 500 metabolic genes, and (h) the same scRNAseq dataset using the corresponding RNA levels of the 10 Met-Flow metabolic proteins.

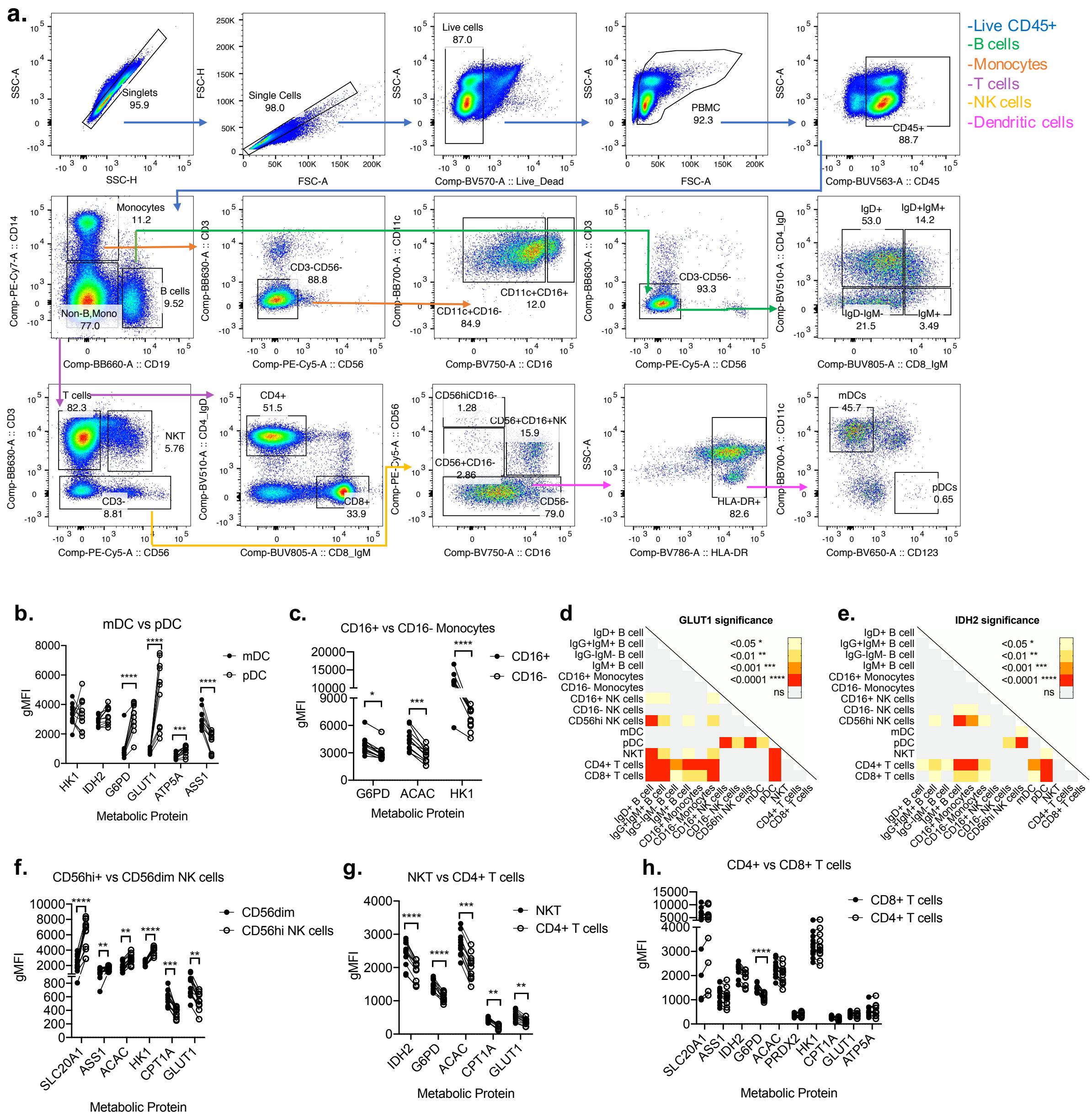

Supplementary Figure 2. Divergent metabolic protein levels across immune subsets. (a) Gating strategy for each immune population. (b) Selected metabolic target expression between mDC and pDCs,(c) and CD16^{+} and CD16^{-} Monocytes. (d) Expression of GLUT1 (e) and IDH2 across cell types. Colors indicate significant p values measured by One-way ANOVA with Friedman multiple comparisons test. (f) Selected metabolic target expression between CD56^{hi} and CD56^{dim} (CD56^{+}CD16^{-}), (g) NKT and CD4^{+} T cells, and (h) between CD8^{+} and CD4^{+} T cells, statistical significance measured by multiple T-test with Holm-Sidak multiple comparisons in n=12 donors.

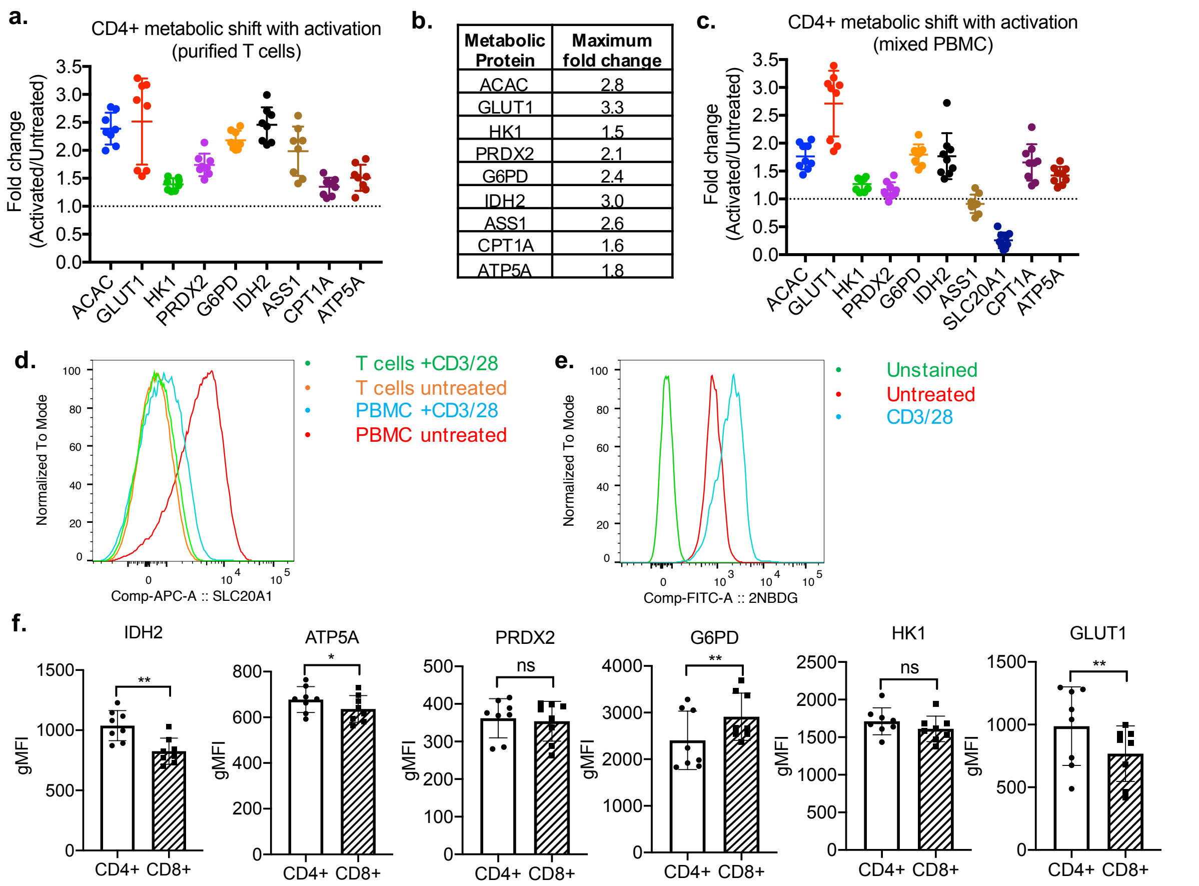

Metabolic Protein	Maximum fold change
ACAC	2.8
GLUT1	3.3
HK1	1.5
PRDX2	2.1
G6PD	2.4
IDH2	3.0
ASS1	2.6
CPT1A	1.6
ATP5A	1.8

Supplementary Figure 3. Comparison of metabolic proteins in T cells (a) Metabolic shift with activation, calculated by fold change of expression of activated over untreated gMFI in CD4+ gated, purified T cells and (b) with maximum fold change values. (c) Expression of metabolic targets in CD4+ T cells from a mixed PBMC population. (d) Expression of SLC20A1 in CD4+ T cells in a mixed sample of PBMC or purified T cells using the same flow cytometry panel. (e) Glucose uptake measurement by 2-NBDG (200uM) in purified T cells by flow cytometry. (f) Comparison of oxidative metabolism and glycolysis associated proteins between CD4+ and CD8+ T cells. Data represents n=8 donors, using paired T-test, Wilcoxon matched signed ranked test.

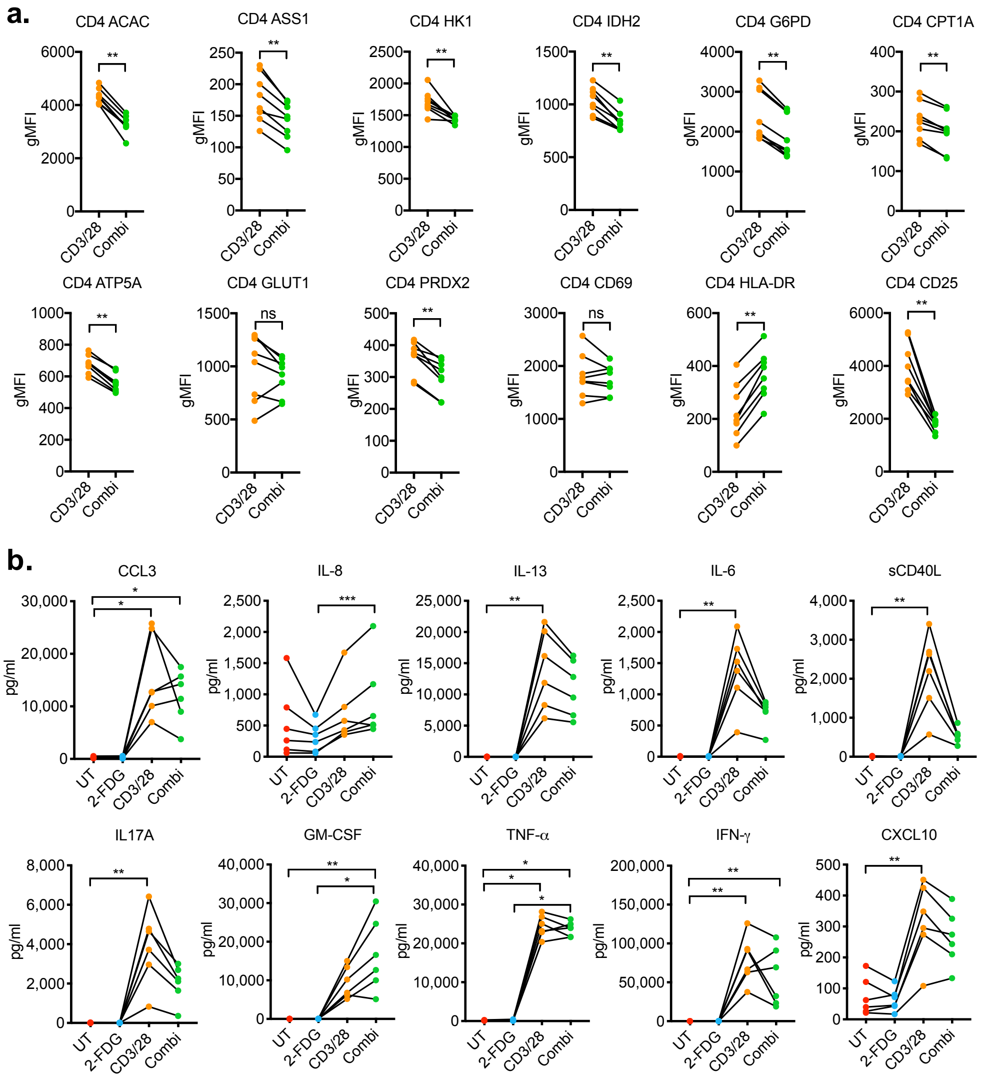

Supplementary Figure 4. Effect of combination (2-FDG+CD3/28) treatment on T cells (a) Expression of metabolic targets between activation (CD3/28) vs. combination (2-FDG+CD3/28) treatment, using paired T-test, Wilcoxon matched signed ranked for analysis. Each dot represents one donor sample, total n=8 donors from 3 independent experiments. (b) Cytokine and chemokine production in supernatants of untreated T cells, 2-FDG alone, CD3/28 alone, Combi (2-FDG+CD3/28). Experiments represent n=6 donors in 2 independent experiments.

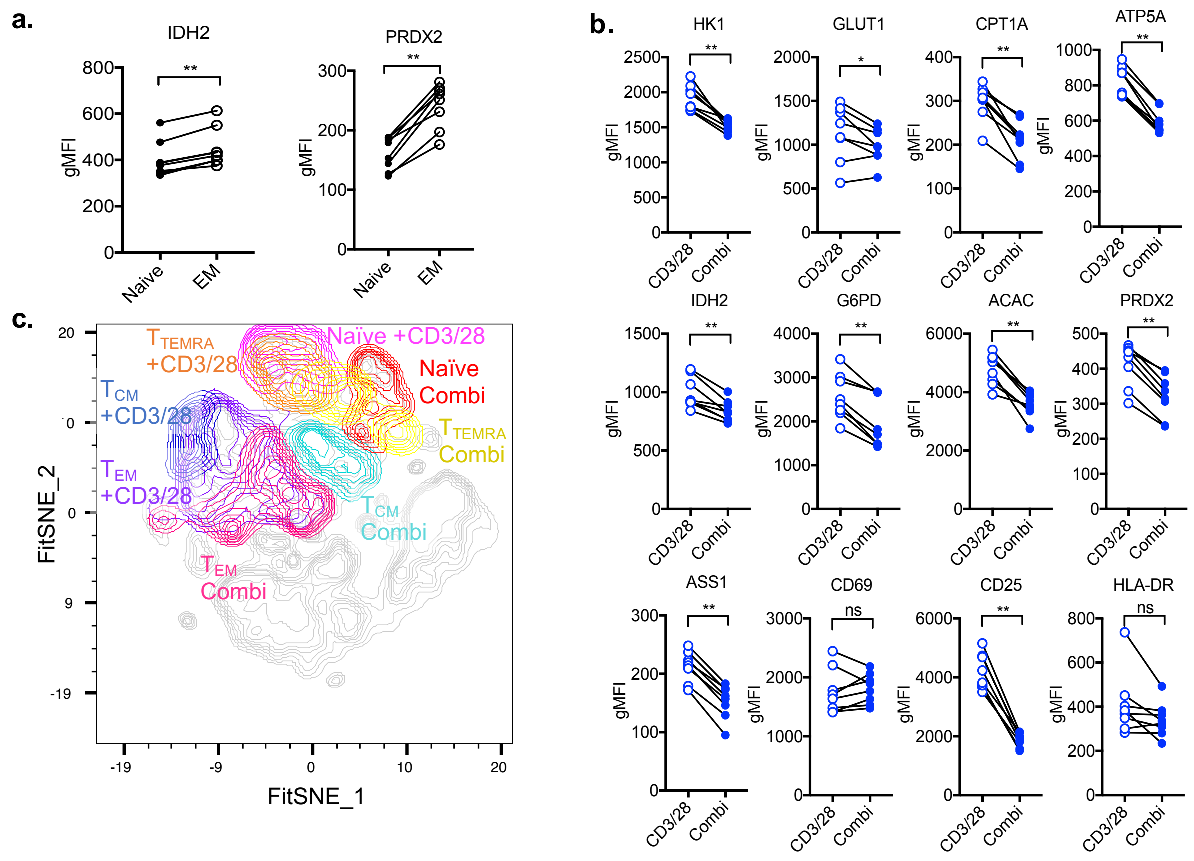

Supplementary Figure 5. Differential expression of metabolic proteins across memory populations (a) Comparison of Naïve vs EM proteins at steady state. (b) Expression level change in T cells with combi (CD3/28 + 2-FDG) treatment in n=8 donors. (c) FitSNE projection of CD3/28 activated and combi treated memory subsets. Data represents n=5 samples.

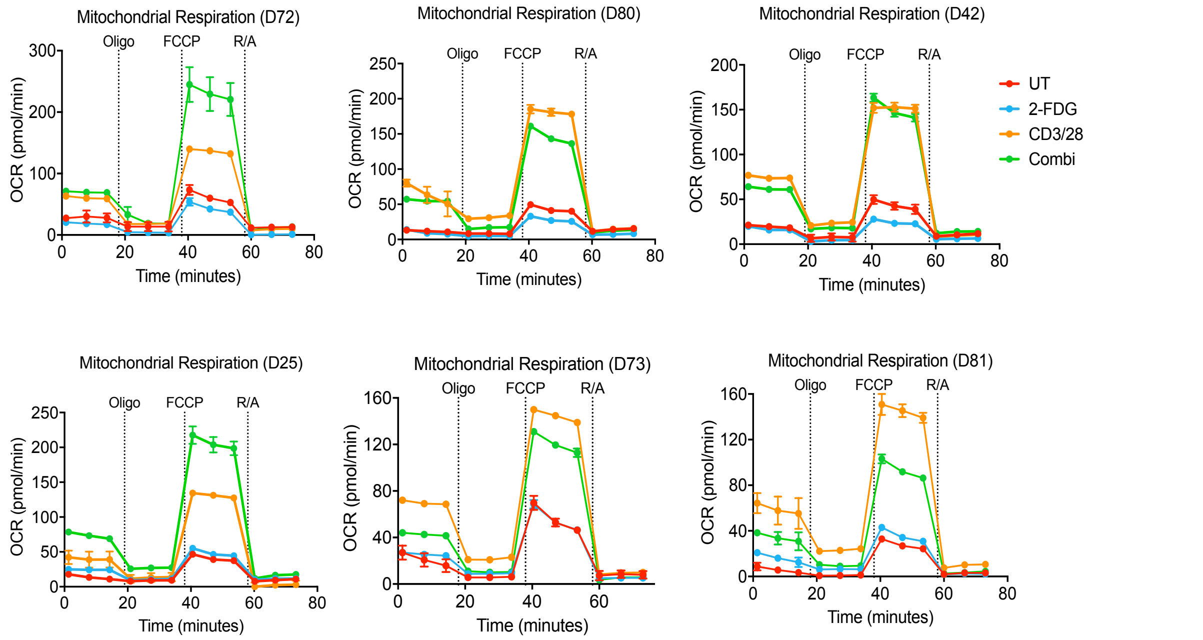

Supplementary Figure 6. Mitochondrial respiration across different donors with treatment Differential mitochondrial respiration rates across untreated, 2-FDG treated, CD3/28 and combi (2-FDG+CD3/28) treated total T cells, each figure represents one donor, in technical triplicates.

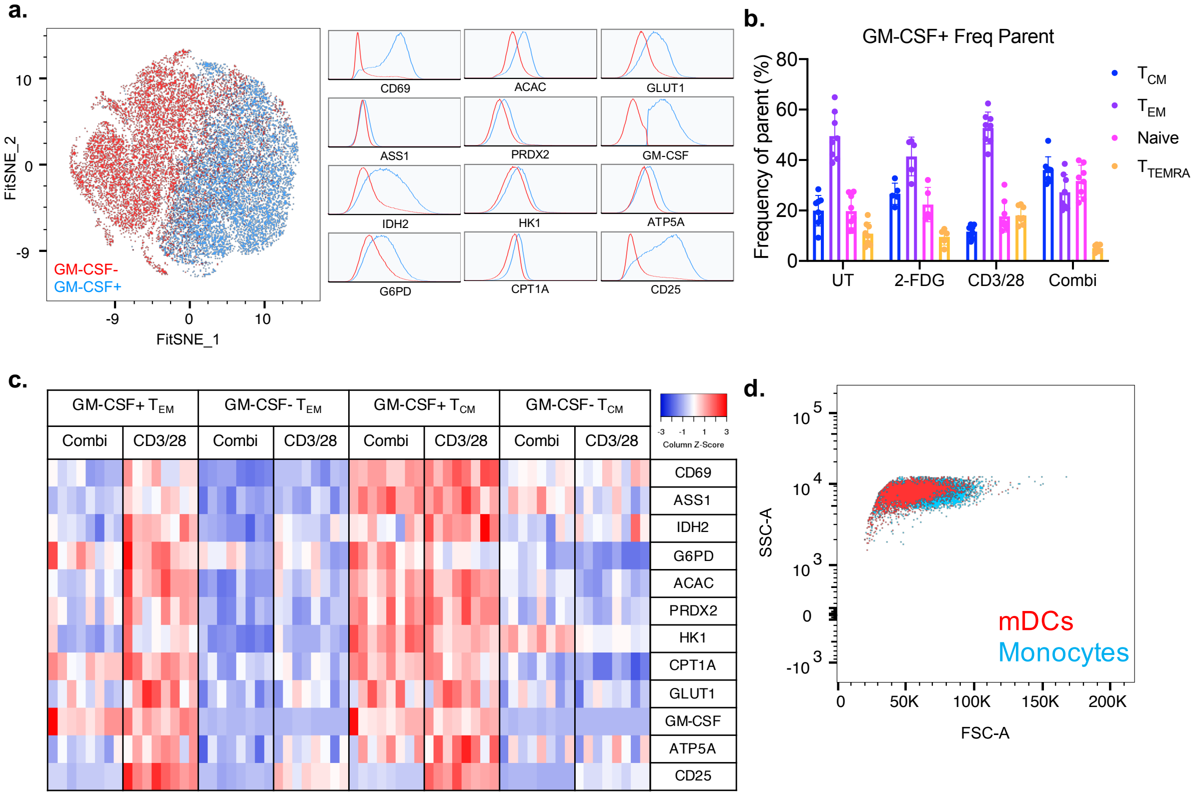

Supplementary Figure 7. Differential metabolic profiles of GM-CSF producing memory T cells (a) Metabolic state of GM-CSF+ and GM-CSF- cells. (b) Frequency of GM-CSF producing cells with treatment. (c) Expression of metabolic targets within the T_{CM} and T_{EM} population, between activated (CD3/28) and combination (CD3/28+2-FDG) treatment in n=8 donors. (d) Forward (FSC) and side scatter profiles of both mDCs and monocyte populations without treatment.
