# Supplementary material for: Met-Flow, a strategy for single-cell metabolic analysis highlights dynamic changes in immune subpopulations
Source: Commun Biol. 2020 Jun 12;3:305. doi: 10.1038/s42003-020-1027-9 (PMC7292829; doi:10.1038/s42003-020-1027-9)
Supplement: Supplementary file 2 — Description of Additional Supplementary Files [file 42003_2020_1027_MOESM2_ESM.pdf]

Description of additional supplementary file

Supplementary Data 1. Source data for the main figures (Excel file).
