# Supplementary material for: Met-Flow, a strategy for single-cell metabolic analysis highlights dynamic changes in immune subpopulations
Source: Commun Biol. 2020 Jun 12;3:305. doi: 10.1038/s42003-020-1027-9 (PMC7292829; doi:10.1038/s42003-020-1027-9)
Supplement: Supplementary file 4 — Reporting Summary [file 42003_2020_1027_MOESM4_ESM.pdf]

nature research

Corresponding author(s): John Connolly

Last updated by author(s): May 11, 2020

Reporting Summary

Nature Research wishes to improve the reproducibility of the work that we publish. This form provides structure for consistency and transparency in reporting. For further information on Nature Research policies, seeAuthors & Referees and theEditorial Policy Checklist .

Statistics

For all statistical analyses, confirm that the following items are present in the figure legend, table legend, main text, or Methods section.

n/a	Confirmed	
☐	☒	The exact sample size (*n*) for each experimental group/condition, given as a discrete number and unit of measurement
☐	☒	A statement on whether measurements were taken from distinct samples or whether the same sample was measured repeatedly
☐	☒	The statistical test(s) used AND whether they are one- or two-sided<br>*Only common tests should be described solely by name; describe more complex techniques in the Methods section.*
☐	☒	A description of all covariates tested
☐	☒	A description of any assumptions or corrections, such as tests of normality and adjustment for multiple comparisons
☐	☒	A full description of the statistical parameters including central tendency (e.g. means) or other basic estimates (e.g. regression coefficient) AND variation (e.g. standard deviation) or associated estimates of uncertainty (e.g. confidence intervals)
☐	☒	For null hypothesis testing, the test statistic (e.g. *F*, *t*, *r*) with confidence intervals, effect sizes, degrees of freedom and *P* value noted<br>*Give P values as exact values whenever suitable.*
☒	☐	For Bayesian analysis, information on the choice of priors and Markov chain Monte Carlo settings
☐	☒	For hierarchical and complex designs, identification of the appropriate level for tests and full reporting of outcomes
☐	☒	Estimates of effect sizes (e.g. Cohen's *d*, Pearson's *r*), indicating how they were calculated

Our web collection on statistics for biologists contains articles on many of the points above.

Software and code

Policy information about availability of computer code

Data collection	FACs data was acquired on a BD X-30 FACSymphony (San Jose, CA) with FACS Diva Version (Version 8.0.1) software
Data analysis	FACs data was analyzed with FlowJo (version 10.5.2) and chord plots was generated using R (version 3.5, circlize, code attached). Other data (e.g cytokine, chemokine) is analyzed with Prism (Graphpad 8.3.1). A two tailed alpha level of 0.05 was chosen for all the statistical tests. Heatmaps were generated using a web-enabled tool named Heatmapper.

For manuscripts utilizing custom algorithms or software that are central to the research but not yet described in published literature, software must be made available to editors/reviewers. We strongly encourage code deposition in a community repository (e.g. GitHub). See the Nature Research guidelines for submitting code & software for further information.

Data

Policy information about availability of data

All manuscripts must include a data availability statement. This statement should provide the following information, where applicable:

- Accession codes, unique identifiers, or web links for publicly available datasets
- A list of figures that have associated raw data
- A description of any restrictions on data availability

Source data for the main figures are included in the Source Data file. Additional data generated and analyzed in this study are available from the corresponding author upon request. The publicly available scRNAseq dataset was published by Zheng and colleagues at https://doi.org/10.1038/ncomms14049.

Field-specific reporting

Please select the one below that is the best fit for your research. If you are not sure, read the appropriate sections before making your selection.

Life sciences study design

All studies must disclose on these points even when the disclosure is negative.

Sample size	Power calculations were not performed for sample size.
Data exclusions	N/A
Replication	Reproducibility was verified by repeating the experiment multiple times and by using different biological replicates each time, i.e. multiple donors.
Randomization	Participants were randomly allocated to this study. Covariates are not relevant, as the study tests the overall concept on the healthy human status.
Blinding	Blinding was not relevant as the study was performed using healthy human donors.

Reporting for specific materials, systems and methods

We require information from authors about some types of materials, experimental systems and methods used in many studies. Here, indicate whether each material, system or method listed is relevant to your study. If you are not sure if a list item applies to your research, read the appropriate section before selecting a response.

Materials & experimental systems

n/a	Involved in the study	
☐	☒	Antibodies
☒	☐	Eukaryotic cell lines
☒	☐	Palaeontology
☒	☐	Animals and other organisms
☐	☒	Human research participants
☒	☐	Clinical data

Methods

n/a	Involved in the study	
☒	☐	ChIP-seq
☐	☒	Flow cytometry
☒	☐	MRI-based neuroimaging

Antibodies

Antibodies used	Metabolic antibodies were purchased from Abcam and custom conjugated by Becton Dickinson (BD) using their fluorochromes, unless otherwise indicated; SLC20A1 (clone EPR11427(2), BD AF647, Abcam ab231703), ACAC (clone EPR4971, BD BUV496, Abcam ab231686), HK1 (clone EPR10134(B), BD BUV661, Abcam ab234112), CPT1A (clone 8F6AE9, BD V450, Abcam ab231704), IDH2 (clone EPR7577, BD BB790, Abcam ab231695), G6PD (clone EPR6292, BD BUV395, Abcam ab231690), GLUT1 (clone EPR3915, Abcam AF488, ab195359), ASS1 (clone EPR12398, BD AF700, Abcam ab231684), PRDX2 (clone EPR5154, BD BUV615, Abcam ab231702), ATP5A (clone EPR13030(B), Abcam AF594, ab216385). BD commercial antibodies used for phenotyping were CD4 (clone SK3, BD, BV480, 566104), CD8 (clone SK1, BD, BUV805, 564912), CD3 (clone UCTH1, BD, BB630, 624294), HLA-DR (clone G46-6, BD, BV786, 564041), CD11c (clone B-ly6, BD, BB700, 624381), CD123 (clone 9F5, BD, BV650, 740588), IgM (clone G20-127, BD, BUV805, 624287), IgD (clone IA6-2, BD, BV480, 566138), CD19 (clone HIB19, BD, BB660, 624295), CD16 (clone 3G8, BD, BV750, 624380), CD14 (clone M5E2, BD, PE-Cy7, 557742),CD56 (clone NCAM16.2, BD, PE-Cy5, 624350), CD45 (clone 2D1, BD, BUV563, 624284), PD-1 (clone MIH4, BD, PE, 557946), ILT3 (clone ZM3.8, BD, BV605, 742807), CD69 (clone FN50, BD, APC-H7, 560737), CD86 (clone 2331/FUN-1, BD, BUV737, 564428), live-dead dye FVS575V (BD, BV570, 565694), CCR7 (clone G043H7, Biolegend, BV650, 353134), CD45RA (cloneHI100, BD, PE, 561883), CD25 (clone 2A3, BD, PE-Cy7, 335789), FOXP3 (clone PCH101, eBioscienceTM, Thermo Fisher, PE-Cyanine5.5, 35-4776-42) and CD14 (M5E2, BD, BV570, 624298).
Validation	Each antibody was validated by the manufacturer, including by western blots. Additionally, this study uses fluorescence-minus-one for experimental controls of each antibody, and is highlighted in the manuscript.

Human research participants

Policy information about studies involving human research participants

Population characteristics	All volunteers were healthy human donors of different age and sex.
Recruitment	Healthy controls from blood services group, HSA
Ethics oversight	National Healthcare Group Singapore, DSRB

Note that full information on the approval of the study protocol must also be provided in the manuscript.

Flow Cytometry

Plots

Confirm that:

☒ The axis labels state the marker and fluorochrome used (e.g. CD4-FITC).

☒ The axis scales are clearly visible. Include numbers along axes only for bottom left plot of group (a 'group' is an analysis of identical markers).

☒ All plots are contour plots with outliers or pseudocolor plots.

☒ A numerical value for number of cells or percentage (with statistics) is provided.

Methodology

Sample preparation	Peripheral Blood Mononuclear Cell (PBMC) were isolated from cone blood of healthy donors using ficoll density gradient centrifugation (Ficoll-Paque, GE Healthcare). Whole blood was diluted in a 1:1 ratio with PBS (GibcoTM, ThermoFisher, 10010023) supplemented with 2 mM EDTA (PBS-EDTA) (InvitrogenTM, ThermoFisher, 16676038). The diluted blood was layered on top of the ficoll in a 2:1 diluted blood to ficoll ratio. The sample was spun at 400 g for 30 min without brake at 21 C. After centrifugation, the PBMC layer was carefully removed and washed twice in PBS-EDTA. Cells were frozen down in freezing medium containing FBS (Hyclone, GE Healthcare, SH30071.03) and 10 % dimethyl sulfoxide (DMSO) at 50x106 PBMC/ml overnight at -80 C and subsequently stored in liquid nitrogen.
Instrument	Samples were acquired on a X-30 FACSymphony (BD)
Software	FACS Diva Version (BD, Version 8.0.1) software
Cell population abundance	T cells were isolated from PBMCs by magnetic separation using CD3 Microbeads (Miltenyi Biotec, 130-050-101), according to the manufacturer's instructions. T cells were tested for purity using flow cytometry and checking CD3+ percentage.
Gating strategy	As indicated in the manuscript gating strategy, the cells were identified as single cells using SSC-H, SSC-A, FSC-H and FSC-A. Subsequently, PBMCs were gated based on FSC-A/SSC-A, excluding all debris which are low in both values. Live/Dead dye was used to then pull out dye negative cells, which are live. And subsequently the cells were gated based on the individual expression of leukocyte markers.

☒ Tick this box to confirm that a figure exemplifying the gating strategy is provided in the Supplementary Information.
